# The effect of propofol on the proliferation and apoptosis of hepatocellular carcinoma cells through TGF-Β1/Smad2 signaling pathway

**DOI:** 10.1080/21655979.2021.1955177

**Published:** 2021-07-29

**Authors:** Zongchao Li, Honglei Liu, Yunxiao Zhang, Hongyu Tan

**Affiliations:** Key Laboratory of Carcinogenesis and Translational Research (Ministry of Education, Beijing), Department of Anesthesiology, Peking University Cancer Hospital & Institute, Beijing, China

**Keywords:** Propofol drugs, transforming growth factor, Smad2 signaling pathway, liver cancer cells

## Abstract

Malignant tumors are a serious threat to human health. Surgical resection is the most effective treatment for liver cancer. However, liver cancer is mostly found at an advanced stage, is difficult to remove by surgery, and has a very high recurrence rate after surgery. The current liver cancer treatment drugs have serious side effects, and the treatment effect is not ideal, far from meeting the clinical needs. Based on this, this paper studies the effect of propofol on the proliferation and apoptosis of liver cancer cells through the TGF-B1/Smad2 signaling pathway, and explores the proliferation, adhesion and apoptosis of cancer cells in patients with propofol. This paper uses a comparative experiment. With medical imaging method, 80 rats with liver cancer in the same period were cultured. High-precision microscope and radiolocation method were used to observe and record the whole process of propofol regulating Smad2 signal pathway. The results show that propofol can effectively inhibit the proliferation of cancer cells in patients with liver cancer. Propofol can increase the activity and content of transforming growth factor-β1 by 12% and 20%, respectively, and then inhibit the proliferation rate of liver cancer cells by 10% through the Smad2 signaling pathway, and exponentially increase the apoptotic number of liver cancer cells. This shows that propofol has a significant inhibitory effect on the cycle of liver cancer cells. Under the action of propofol, the life cycle of liver cancer cells is shortened, which provides a certain theoretical basis for the treatment of liver cancer.

## GRAPHICAL ABSTRACT

**Figure:**
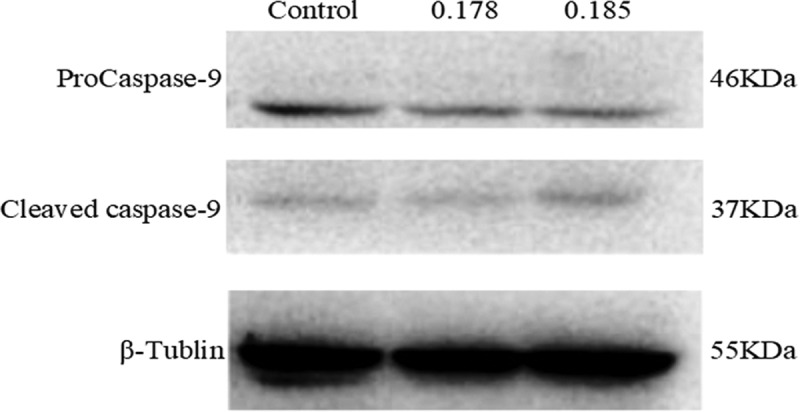

## 1. Introduction

Hepatocellular carcinoma (HCC) is the most common primary HCC. It has an early onset and usually progresses once symptoms appear. HCC lacks response to TGF, which can cause TGF to fail to inhibit cell proliferation and induce cell apoptosis, which leads to the occurrence of tumors, and the incidence and mortality of liver cancer are increasing year by year. Therefore, exploring the regulation mechanism of the occurrence and development of liver cancer will not only help to improve the molecular network of liver cancer, but also help discover new markers of liver cancer and develop new drug targets, which have important clinical significance clinical diagnosis and treatment of liver cancer.

Transforming growth factor-(TGF) is a key inflammatory regulator for liver diseases from hepatitis to cirrhosis to liver cancer [1]. In the carcinogenic stage, TGF-β changes from a tumor suppressor to a carcinogen, thereby promoting the development of liver cancer [2]. As an important cytokine in the microenvironment of chronic liver disease, TGF-the molecular network that promotes the progression of HCC has not yet been fully elucidated. TGF-P and TGF-β/Smad signaling pathways play a key role in the pathogenesis of liver fibrosis [3].

In order to explore the effect of propofol on the proliferation and apoptosis of liver cancer cells through the TGF-β1/Smad2 signaling pathway, detailed experimental analysis was carried out. In the article, Li conducted a detailed study on how to inhibit the proliferation of liver cancer cells, analyzed various medical drugs and chemicals, and found that propofol drugs have an excellent inhibitory effect on the proliferation of liver cancer cells [4]. Sun conducted a follow-up survey on a number of liver cancer patients, and tracked the TGF-β1 release-related factors with radioactive pigments through the immune tracking method, and found that this channel does have an effect on the proliferation of liver cancer cells [5]. Cui found through related studies that the various channels of Smad have a great correlation with the changes and proliferation of liver cancer cells, when the shad channel is active; the proliferation rate of liver cancer cells is significantly reduced [6]. Wang mentioned in the article that the propofol drug propofol can proliferate and apoptosis of liver cancer cells through the TGF-β1/Smad2 signaling pathway, and the effect is very obvious [7]. Xu found through experiments that taking a certain dose of propofol can effectively inhibit the proliferation of cancer cells in patients with liver cancer, propofol can increase the activity and content of transforming growth factor TGF-β1 in patients, thereby inhibiting liver cancer through the Smad2 signaling pathway cell proliferation rate [8].

This article mainly studies the effect of propofol on the proliferation and apoptosis of liver cancer cells through the TGF-β1/Smad2 signaling pathway. It is the first time to use propofol to treat liver cancer patients. This new type of drug has a very obvious inhibitory effect on liver cancer cells, and the therapeutic effect is excellent. Secondly, this article uses a high-precision microscope to investigate in detail the path and direction of the drug in patients with liver cancer, record and observe the proliferation, adhesion and apoptosis of cancer cells in the patient’s body, and use modern measuring instruments to study and study in detail at the molecular level. Track related reaction mechanisms and effects. Compared with the past, the accuracy and reliability of the experiment are greatly improved.

## 2. Material and methods

### 2.1. Precancerous lesions and initial stage of liver cancer

HCC comes from precancerous lesions, such as hepatocyte regeneration nodules, low-grade and high-grade malignant hyperplastic nodules [9]. It is believed that the accumulation of gene mutations in precancerous nodules is an important cause of HCC and is usually related to a specific etiological basis. HCC gene mutations involve four most important pathways: the p53 pathway involved in DNA damage repair, the TGF-pathway involved in proliferation inhibition, the WNT pathway involved in cell connectivity and signal transduction, and the Rb1 pathway regulation involved in the cell cycle [10]. The stimulating factors produced by chronic inflammatory cells in the liver environment promote the regeneration of liver cells, and together with carcinogens and viruses cause these genetic disorders. For example, in the case of HBV virus infection, the Hobs protein binds to the tumor suppressor gene p53 and inhibits its function. The integration of the viral genome and the host genome may also lead to stable structural changes in the genome, such as gene mutations, gene rearrangements, and DNA strand breaks. In chronic hepatitis and liver cirrhosis tissues, the proportion of DNA methyltransferase increases, which indicates that changes in methylation levels, may be related to precancerous lesions [11]. These factors accumulate in liver cells and jointly promote the malignant transformation of cells. In the occurrence of liver cancer, alleles are usually absent or increased, and the resulting genomic instability promotes the activation or inactivation of oncogenes or tumor suppressor genes. For example, it was found that the tumor suppressor gene CDKN2A on chromosome 16Q and the proto-oncogene C-MYC on chromosome 8Q both have structural changes of liver cancer. These changes are usually caused by micro environmental stimuli, such as afflation B1 and the G/T mutation in codon 249 of the tumor suppressor gene p53. Oxidative damage in the inflammatory environment can lead to mutations in the mitochondrial genome. Genetic changes lead to the formation of malignant hepatocyte populations and eventually induce HCC [12,13].

In liver cancer tissues, all components except liver cancer cells are collectively referred to as stromal, including hepatic stellate cells, peripheral lymphocytes, chuffer cells, my fibroblasts, epithelial cells, immune cells, tumor blood vessels and lymphatic components, and are tissue-specific stromal cells and their expression metabolites [14,15]. In the microenvironment of liver cancer, there is an interaction between cancer cells and stromal, which can affect the whole process of cancer cell development and enhance or inhibit the development of liver cancer. Important proteolysis enzymes in liver cancer mainly include matrix metalloproteinase (MMP) and urokinase-type plasminogen activator (UPA). These hydrolases degrade the matrix and create conditions for tumor cell infiltration and metastasis and the formation of new blood vessels. The overexpression of MMP-2 and MMP-9 affects the metastatic ability of liver cancer [16]. When MMP is activated by transport-related molecular pathways and the expression of tissue inhibitors of metalloproteinase (TIMPs) remains unchanged, the MMP/TIMP balance will be disrupted. TIMP cannot fully play its role in inhibiting tumor cell invasion and metastasis and the formation of new blood vessels, thereby affecting the stability of the matrix [17]. E-cadherin (E-cadherin) is a classic member of the Cadherin family. It is a calcium-dependent glycoprotein that mediates the formation of cell adhesion and tight junctions. Its reduced expression and gene mutation are conducive to tumor proliferation, invasion and metastasis [18].

In normal liver cells, E-cadherin usually forms an intracellular adhesion complex (cadherin-catenin complex) with -catenin, which is distributed at the junction of cells under the membrane to maintain normal adhesion and signal transduction guide [19]. When the expression of E-cadherin is lost in the cell membrane, catenin cannot be positioned correctly in the sub membrane, but is transferred to the cytoplasm or nucleus. It is related to epithelial-mesenchyme transition, dedifferentiation, vascular infiltration and clinical prognosis. New angiogenesis is a necessary condition for liver cancer metastasis. When insufficient blood circulation leads to hypoxia, vascular endothelial growth factor (VEGF) promotes the formation of new blood vessels, and its expression is related to the metastasis and prognosis of liver cancer [20].

### 2.2. Important molecular pathways and genes of liver cancer

Lack of cellular response in cancer of TGF can cause TGF-can not inhibit proliferation and cause apoptosis, which leads to cancer. However, in HCC, the TGF-downstream mutations Smad2 and Smad4 are less than 10%. Overall, the classic SMAD-dependent TGF pathway shows only a 25% change in liver cancer. Excessive activation of downstream SMAD-independent pathways (such as PI3K/Act and Raps/MAPK) may be the main reason for the transformation of TGF-β from a tumor suppressor into carcinogenesis. A multifunctional protein called -catenin, as a membrane protein, it can combine with E-cadherin to form a complex and participate in the formation of cell connections [21]. As an effector molecule downstream of the WNT pathway, -catenin is involved in tumorigenesis induced by the WNT pathway. Phosphorylation of GSK-3 downstream of WNT signal deregulation cannot normally degrade-catenin. The transport of accumulated catenin to the nucleus leads to the transcriptional activation of specific genes, including C-MYC, cycling D1, etc. When a mutation occurs in the gSK-3 targeted amino acid on -catenin, -catenin cannot be degraded normally, so it is transported to the nucleus. The disordered state of -catenin pathway may be related to the induction of HCC stem cells and the occurrence of HCC. The promotion of liver cancer metastasis may be due to its induction of epithelial-mesenchyme transition [22].

In liver cancer, the expression of PTEN gene is usually reduced, deleted or mutated, and various minas that target the PTEN gene are also up-regulated. The low expression of PTEN is usually related to liver cancer grade and poor prognosis. In the mouse PTEN knockout model, atypical hepatocytes are formed, which are usually markers of precancerous lesions [23]. The possible mechanisms of PTEN as a tumor suppressor gene include antagonizing PI3K/Act pathway to resist apoptosis, promoting proliferation and promoting cancelation. Maintain chromosome stability and participate in DNA damage repair, stable E-cadherin/-catenin complex and cell-to-cell connection. The p53 gene is one of the earliest tumor suppressor genes discovered. It has the functions of stabilizing chromosomes, promoting cell differentiation and senescence, and controlling cell proliferation [24]. Genes are divided into wild type (WT-p53) and mutant type (MT-p53). Wild-type P53 is mainly used to inhibit cell growth and induce apoptosis. When mutations occur, they stimulate and promote cancer cells.

### 2.3. The mechanism of TGF-β1/Smad2 signaling pathway in tumor pathology

TGF-P participates in many aspects of the injury response and plays a very important role in regulating tumor growth, fiber formation, axing disorder and death [25]. TGF-β interferes with the process of many diseases in the liver, which is not only closely related to liver fibrosis and cirrhosis caused by chronic liver injury, but also related to the occurrence of HCC. In normal unstipulated tissues, the basal level of TGF-P is continuously secreted, enough to ensure homeostasis. However, when the tissue is damaged, in order to control the inflammation and proliferation of regenerative cells, a large amount of TGF-P is produced from the blood and continuously released. A similar mechanism occurs during cancer. The TGF-P signaling pathway in the liver plays an important role in chronic inflammation and fibrosis. First, the liver damage related to cell tissue caused by chronic inflammation causes it to produce TGF-ply, and at the same time, activate liver cells. Stellate cells (HSC) are transformed into my fibroblasts through a transformed form MF is transformed into muscle cells in a timely manner, depending on the interpretation of anticrime or paracrine and TGF-ply [26]. My fibroblasts are under the action of TGF-PL It produces a large amount of collagen and fibrinogen, which eventually leads to the appearance of liver fibrosis. In addition, TGF-PL can stimulate the body’s immune system, thus reducing the inflammatory response of the arrhythmia and the ongoing state of chronic liver damage and ultimately accelerating the liver. On the other hand, the occurrence and development of liver cancer are also closely related to TGF-PL. TGF-PL plays a variety of roles in the development of liver cancer, including the regulation of uterine tumor, vascular permeability, and interaction between newborns. The effect, the spread of cancer cells, etc. It is interesting that based on the results of various studies. TGF-P has two different effects in the pathogenesis of liver cancer. The expression of TGF-P in a variety of tissue cells can inhibit tumors. However, liver cancer still has an anticrime TGF-P loop while retaining the sensitivity of the tumor suppressor effect, and it is functional. As for the specific mechanism of this autocuing cycle, current research cannot fully explain it. Experiments have found that in the chronic inflammation process in the liver, promoting the development of liver cancer, the TGF-P signaling pathway plays an important role in the nucleus. First, the inactivation of TGF-P is beneficial to tumor cells and helps reduce it inhibit cell proliferation, thereby affecting the severity and invasion ability of the liver. Secondly, liver cancer cells develop an autocuing TGF-P pathway to improve the secretion of inflammatory cytokines, growth factors and chemokine’s, etc. for tumor cell proliferation and the transfer provides the environment

The progression of cancer is also related to the involvement of TGF-P. TGF-P may promote liver cancer metastasis, including promoting epithelial transformation and accelerating the invasion of cancer cells. As the most important mediator of TGF-PL, the Smads protein can transmit signals to the cytoplasm of this signaling pathway in the nucleus. The T-pi/Smad signaling pathway achieves signal transmission through two types of pathways, classic and non-classical. In the classic TGF-pl/Smad signaling pathway, T-pi enters the type II receptor (QRII) that binds to the cell surface, phosphorylates the C-terminus of Smad2 and Smad3; while in the non-canonical signal pathway, it is through various killer-activated protein kinase (Mitogen-activated protein kinase, MAPK) pathways, carbonating the junction region of Smad2 and Smad3 (Linker region, L). These two pathways together produce different phosphorylation subtypes: C-terminal phosphorylated Smad2/3, linker region phosphorylated Sma/3 W and double acidified Smad2/3. Among them, invades L/C to transmit fibrosis signals, pSmad3C transmits growth inhibitory signals, and pSmad3L transmits cancer signals. The main reason for liver fibrosis to develop into liver cancer is that the growth inhibitory signal of psmad3C is transformed into the pro-fibrosis signal of the cancer-promoting signal of pSmad3L. The above-mentioned Smad protein binds to Smad4 to form a smAD2/3/4 complex, which is transported to the nucleus by the cytoplasm through nuclear transport receptors Lmp7 and Lmp8, thereby regulating the transcription of king gene. On the other hand, Smad7 plays a feedback inhibitory role in the TGF-pySmad signaling pathway.

## 3. Results

### 3.1. Selection of Experimental Objects and Materials Needed for Experiments

Eighty 8-week-old female C57BL/6 mice were selected and purchased from the Scientific Laboratory Animal Center, with an average weight of 20 grams. The patients were randomly divided into four groups: 20 patients in each group 1 Control group (control group)-intraperitoneal injection of phosphate buffered saline (PBS, pH 7.4). Bacterial lipopolysaccharide group (LPS group)-intraperitoneal injection of 5 mg/kg bacterial lipopolysaccharide. Propofol group: intraperitoneal injection of protocol 10 mg/kg, CLPS + Prop (bacterial lipopolysaccharide + protocol)-intraperitoneal injection of 5 mg/kg bacterial lipopolysaccharide and 10 mg/kg isopropyl acetone. Keep all animals in the same cage every five times. The day-night cycle keeps the room temperature at 25°C. The procedures for purchasing, breeding and testing laboratory animals have been approved by the Laboratory Animal Center of Southern Hospital. The materials required for the experiment are shown in Table 1.

**Table 1.** The materials needed for the experiment

Name of the material	Purchasing company
Diisopropyl phenylkul	Sigma
Lipopolysaccharide	Escherichia coli
The PrimeScript RT Reagent kit	TaKaRa
Premix Ex TaqTM II kit	TaKaRa
Plasmid extraction kit	Promeg

Isopropyl (2, 6-diisopropyl) and bacterial lipopolysaccharide (from E. coli 055: B5) were purchased from Sigma (USA). RNA reverses transcription kit (Prime Script RT kit, DRR037A) and real-time fluorescent quantitative PCR kit (SYBR Premix Ex Tat II Kit, DRR820A) were purchased from Taker (Japan). Other chemical reagents are of pharmaceutical grade, all from reagent suppliers.

### 3.2. Experimental Cell Culture

Human liver cancer cells (HepG2) and human acute mononuclear leukemia cells (THP-1) were purchased from the Culture Collection (Manassas, VA, USA). HepG2 cells grow at 37 years of age. C, 5% CO_2_ and DMED medium containing 10% newborn fetal calf serum were incubated for static culture, and logarithmic growth phase cells were taken for experiments. Thp-1 cells were cultured in RPMI-1640 medium at 37°C, 5% CO_2_ and 10% neonatal fetal calf serum. The logarithmic growth phase cells were used in the experiment. Before each experiment, 5% CO_2_. 37 phorbol 12-myristate 13-acetate (PMA) was used. Thp-1 cells were incubated in C for 72 h and induced to differentiate into macrophages. The cells were adhered to a 6-well plate, a 12-well plate or a 60 mm petri dish, and processed when the cells grew to 80–90% confluence.

Before the experiment, PBS was washed twice, and antibody-free serum-free PBS was washed for the last time. DMEM medium: Take a bag of DMEM medium dry powder, dissolve it in 800 ml of double-distilled water, and then add 1.5 g NaHCO3 to adjust the pH to 7.0 to 7.2. Then, 1 moll-I sodium pyruvate and 2 mmol-L-1 glutamine were added, and the double steam water was stabilized to 1 L. The samples were filtered and sterilized, and stored at −20°C. Add double anti-liquid (final concentration: penicillin 100 U/ml, streptomycin 100 U/ml). TGF-Pi: Use sterile 4mmoH/chill containing 0.1% bovine serum albumin (BSA) to prepare stock solution, and −2 (RC storage for storage). MAPK pathway specific inhibitor: ERK inhibitor PD98059, JNK inhibitor SP600125 and P38 inhibitor SB203580 were dissolved in DMSO, and then separated (the final concentration of DMSO is less than 0.1%), and stored in the dark at −20°C.

### 3.3. Experimental Method

The experiment was divided into blank control group, TGF-Pi model group, ERK inhibitor group, JNK inhibitor group and P38 inhibitor group. HSC cells in the logarithmic growth phase were seeded into a 24-well plate containing 10% NBS in DMEM to adjust the cell density of 5xL06i~I, and seeded into a 24-well plate with a sterile glass slide. Incubate at 37°C in CO2 with saturated humidity, and incubate until the length of HSC cells adhering to the wall reaches 30% of the monolayer. Forty percent of the serum-free DMEM synchronized cells were replaced and cultured for 24 hours. Five hours before the termination of the culture, ERK inhibitor (PD98059) and 10 timed/L were added to the ERK inhibitor group, JNK inhibitor group and P38 inhibitor group respectively. JNK inhibitor (SP600125) or 10^111,101 and some 1538 inhibitors (SB203580), the final concentration of 40 polo [TGF-PI] was added to groups other than the blank control group before terminating the culture. At the end of the training, the cellular immune combustion light staining method was used to detect the effect of the three MAPK inhibitors TGF-PI on the induction of HSC Shads protein transfer and nucleation in cells. Give up the culture, fix 4% formic acid polymer within 15 minutes, 4 incubate the cells with primary antibody (1:50) at 4°C for 30 minutes, incubate with FITC (1:100) at 37°C for 1 hour, and incubate with DAPI (1:100) for 10 minutes at room temperature. Seal 80% glycerin and observe under a burst microscope.

In addition, the experiment was divided into blank control group, TGF-Pi model group, ERK inhibitor group, JNK inhibitor group and P38 inhibitor group. HepG2 cells adhered to 90% of the monolayer membrane and 95% of the cells were trypsin zed, and the cell density was adjusted with DMEM containing 10% NBS. Inoculate the cells in a 24-well plate with a sterile glass slide, and culture under 5% CO_2_ 37°C saturation. HepG2 cells adhere to 30% of the wall length. Forty percent of the serum-free DMEM synchronized cells were replaced and cultured for another 24 hours. 5 hours before the end of the culture, add 10%fooH/IP38 to ERK, JNK and P38 inhibitors inhibitor (SB203580) group. Before the end of the culture, add TGF-Pi at a final concentration, and set up three compound wells in each group. At the end of incubation, the localization of phosphorylated Smad3C and Smad3 was detected by fine Stiff immunofluorescence staining.

### 3.4. Experimental Result Detection Method

1. Cell proliferation experiment

After regular digestion of SK-Hep1 and Huh7 cells, take a sample with a cytometer, and then measure the cell concentration with a cytometer. According to the experimental grouping, each group has two replicate wells, and 100 ul of cell suspension is added to the 96-well plate. The number of cells is 8000 (SK-Hep1) and 12,000 (Huh7) respectively. Every 24 h, different concentrations of propofol (0.12 μmol/L, 0.178 μmol/L, 0.185 μmol/L) were given for treatment, and two drugs were added, and the treatment time was 48 h. After 48 h, discard the culture medium, dilute Edu solution (reagent A) 1000 times with MEM medium containing 10% FBS, add 100 μl Edu diluted solution to each well, and put it in a 37°C, 5% CO2 cell incubator. For the doubling time, SK-Hep-1 was incubated for 2 h, and Huh7 was incubated for 6 h. After incubation, discard the culture medium and wash the cells twice with 200ul PBS, 5 min/time static. Add 100 μl of 4% paraformaldehyde in PBS, and let stand at room temperature for 30 min. Discard the fixative solution 4% paraformaldehyde in PBS, add 100 μl of 0.2% glycine solution, shake for 5 minutes on a shaker, and discard the glycine solution. Add 100 μl of PBS, shake for 5 min. Discard PBS, add 100 μl 0.5% TritonX-100, and incubate for 10 min on a shaker. Discard 0.5% TritonX-100 and wash with PBS for 5 minutes. Add 100 μl of 1× Apollo staining reaction solution (see Table 1 for the preparation method), put the 96-well plate in the lunch box, and incubate for 30 minutes in a decolorizing shaker at room temperature, protected from light. Discard the staining reaction solution, add 100 μl 0.5% TritonX-100 liquid, and wash twice with a decolorizing shaker, 10 min/time. Discard the penetrant, add 100 μl methanol to wash for 5 min. Discard the methanol and wash with PBS for 5 min. Dilute reagent F (Hoechst 33,342) 100 times with deionized water, add 100 μl of Hoechst 33,342 diluent, and incubate for 30 min at room temperature, protected from light, and on a decolorizing shaker. The staining reaction solution was discarded, and 100 μl PBS was added to wash twice, 5 min each time. Images were acquired under a fluorescence microscope, five fields of view were randomly selected for each well, and the number of cells was counted with IPWIN60 software.

1. Flow Cytometer AnnexinV-FITC/PI Method for Measuring Apoptosis

After regular digestion of SK-Hep1 and Huh7 cells, the cells were inoculated into groups according to the experiment. After 24 hours of culture in a 37°C, 5% CO2 cell incubator, the final concentration of each group was 0.12, 0.178, and 0.185 μmol/L according to different concentrations of propofol. After 24 hours of treatment, the cell culture medium was collected into a centrifuge tube, K-Hep1 and Huh7 cells were routinely digested, and the cell suspension was transferred to the same centrifuge tube again. Centrifuge at 1000 rpm for 5 min, and discard the supernatant. Add 300 μl of pre-chilled 1× AnnexinVBindingBuffer and blow off the cell pellet, transfer the stock solution to a 1.5 ml EP tube, add 5 μl AnnexinV-FITC and 5 μl PI, shake gently to mix. Put the EP tube on ice and protect it from light, and immediately use flow cytometry to detect and analyze.

1. Cell cycle experiment

After regular digestion of SK-Hep1 and Huh7 cells, the cells were inoculated into groups according to the experiment. After regular digestion of SK-Hep1 and Huh7 cells, the cells were inoculated into groups according to the experiment. After 24 hours of culture in a 37°C, 5% CO2 cell incubator, the final concentration of each group was 0.12, 0.178, and 0.185 μmol/L according to different concentrations of propofol. After acting for 24 h, K-Hep1 and Huh7 cells were digested routinely, and the cell suspension was transferred to a centrifuge tube. Centrifuge at 800 rpm for 5 min, and discard the supernatant. Wash the cells with pre-cooled PBS, centrifuge at 800 rpm for 5 min, and discard the supernatant. Add pre-cooled 70% ethanol to the centrifuge tube and fix at 4°C overnight. Centrifuge at 2500 rpm for 10 minutes and discard the supernatant. Wash with PBS for 5 minutes, centrifuge at 800 rpm for 5 minutes, and discard the supernatant. Add 300ulPI working dye solution to the centrifuge tube, then transfer to the EP tube, incubate in the dark for 20 minutes, and then perform flow analysis.

### 3.5. Experimental Data Processing and Algorithms

The experimental data uses SPSS statistical software. In addition, in order to improve the accuracy, the relevant monitoring sensors are used for error correction and analysis on the basis of the software. The algorithms required for statistical experimental data are shown in formulas 1 and 2.

(1) $$SM_{u}=X_{G}+X_{I}+X_{I}*X_{G}$$

(2) $$M_{S}=SM/(X-1)$$

Among them: SM is the average sum of the samples, MS is the variance sample difference.

## 4. Discussion

### 4.1. Analysis of the Effect of Propofol on the Proliferation and Apoptosis of Liver Cancer Cells

**Figure 1.**
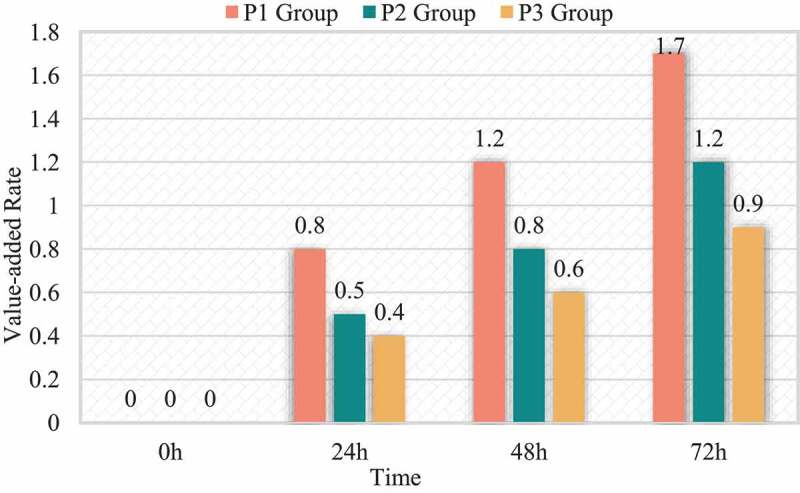
Relationship between proliferation rate and isopropyl dose

Comparing the growth rate of HCC cells HepG2 in each group, it was found that the difference in the growth rate of isoproterenol at different therapeutic concentrations was statistically significant. The proliferation rate of P2 and P3 group was lower than that of P1 group (p < 0.05), the proliferation rate of P2 group was >0.03. The proliferation rate of group 3 (P < 0.05). The differences in the proliferation rate of parasitic vinegar between the groups at different treatment times were statistically significant. There is an interaction between isopropanol age treatment concentration and propofol treatment time (I = 433.681, P = 0.000). The reproduction rate of P1, P2, P3 treatment for 72 hours> the reproduction rate of treatment 48 h> the reproduction rate of treatment 24 h (I < 0.05). The proliferation rate of the P1, P2 and P3 groups treated for 72 h; the proliferation rate of the I, P1, P2 and P3 groups treated with >48 h; the proliferation rate of the I group treated with >24 h (p < 0.05); similar to the C sisters in comparison, the proliferation rate of HepG2 cells in the P1, P2 and P3 groups decreased at each treatment time, while the proliferation rate of HepG2 cells in the I group increased at each treatment time (P = 0.41). The relevant data is shown in Table 2 and Figure 1.

**Table 2.** Effects of amino acids on protein synthesis rate in human muscle

Isopropyl dose	Tumor cell proliferation rate of HepG2 cells	Processing time
0.12 ml	1.565*10^7^/s	0.5
0.178 ml	2.35*10^7^/s	1.25
0.185 ml	3.21*10^7^/s	1.35

**Figure 2.**
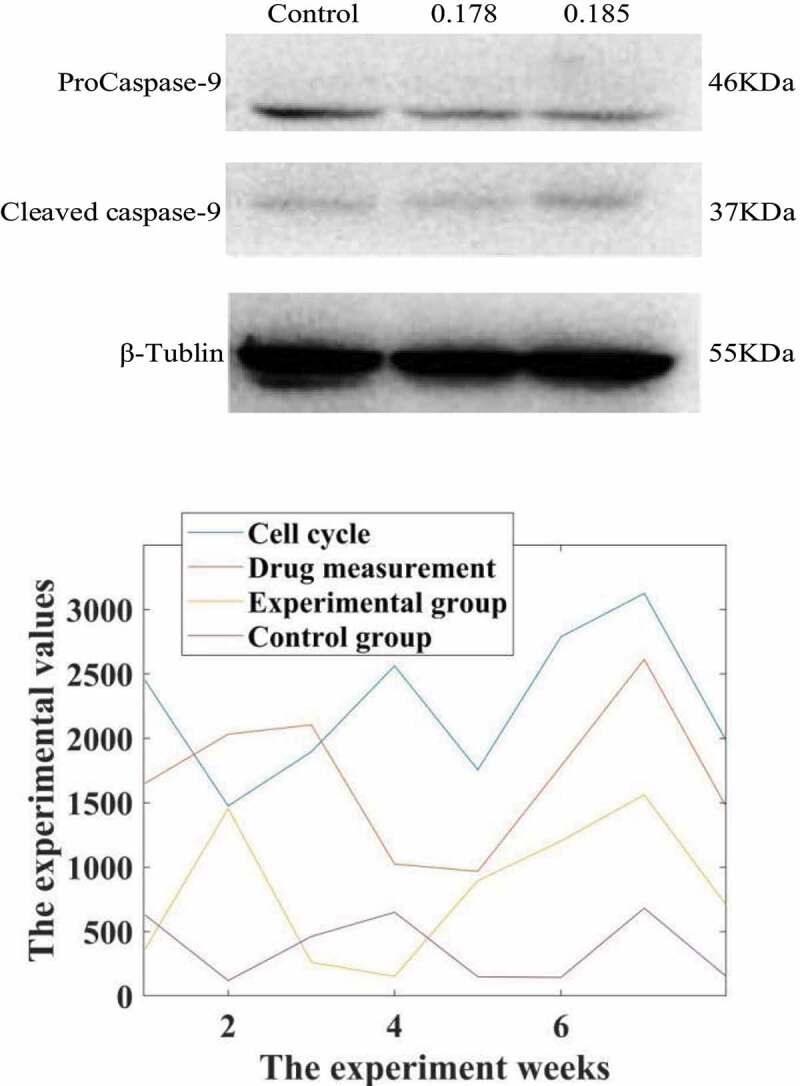
Western-Blot detection of the effect of propofol on the apoptotic protein Caspase-9 in liver cancer cells. Intervention of propofol on hepatocellular carcinoma cell cycle

After 0.178 and 0.185 μmol/L propofol acted on liver cancer cells 24, Western-Blot experiment found that compared with the control group, propofol significantly reduced the expression of the apoptotic protein ProCaspase-9 protein and increased the protein level of Cleaved Caspase-9. The expression is dose-dependent, and the results are shown in Figure 2.

Studies have shown that the comparative cell cycle diagrams of each group of HepG2 cell cycles show that the cell distribution ratios in G1, G2/M and S phases have not changed significantly. Compared with the C group, the G1, G2/M and S phase cell distribution ratios of the I, P1, P2 and groups were not statistically significantly different (the test statistics were F = 8.123 and F = 0.087, respectively). F = 0.720, P = 0.552; F = 14.107, P = 0.092), the result is shown in Figure 2.

It can be seen from the data in Figure 2 that propofol has a significant inhibitory effect on the cycle of liver cancer cells. Under the action of low doses of propofol drugs, the life cycle of liver cancer cells is reduced, which is more than that of normal cancer cells. In contrast, the life cycle of cancer cells in rats treated with propofol drug intervention was reduced by 85.73%.

**Figure 3.**
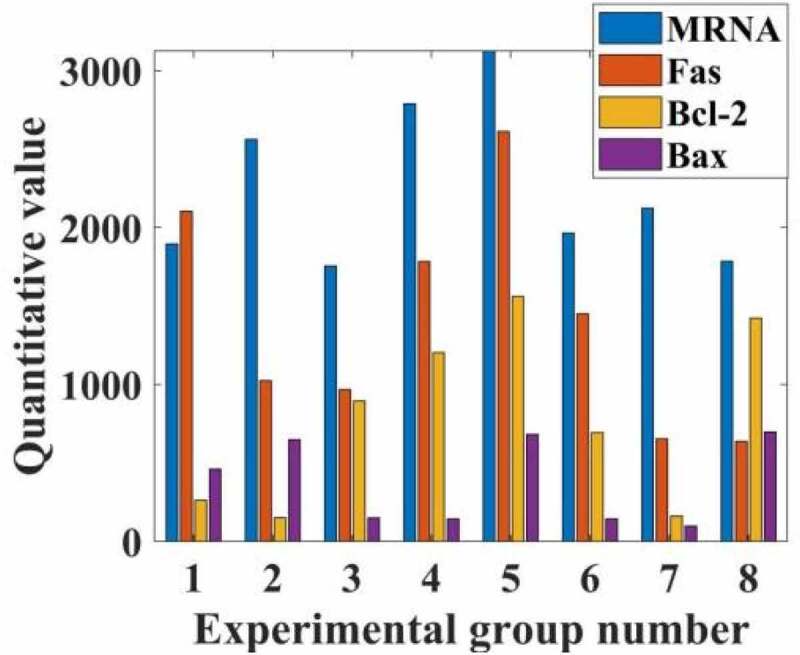
Inhibitory rate of propofol on the rate of mRNA division of hepatocellular carcinoma cells in patients with hepatocellular carcinoma

The expression of Fas, Bcl-2, Box and Box mRNA in HepG2 cells of each group was compared. Compared with group C, the relative expression of PI3, Fas, BD-2 and Box mRNA in HepG2 cells was up-regulated (P < 0.05). The relative expressions of Fas, Bcl-2 and Box mRNA in the experimental group were not statistically significant (respectively = 0.551; I = 1.000; P = 0.448); compared with the PI group, the expression was up-regulated (P < 0.05). Compared with the experimental group, the expressions of Fas, Bcl-2, Box and mRNA in the control group were up-regulated (I = 0.000; P = 0.000 respectively); there was no statistically significant difference between the Box and Bcl-2 mRNA ratio groups (nursing 1.856, P = 0.150), the relevant data is shown in Figure 3.

From the data in Figure 3, it can be seen that propofol can greatly reduce the proliferation rate of liver cancer cells by adjusting the rate of mRNA division of liver cancer cells in liver cancer patients, and by inhibiting and slowing down the expression of Fas, Bcl-2, and Bax of mRNA. Compared with the normal group, the propofol drug can reduce its proliferation rate by 74%, and the number of liver cancer cells was reduced by 35 percentage points compared with before the drug.

*4.2. The Effect of Isopropanol on the Growth of Hepatocarcinoma Cells and the Effect of TGF-Β1/Smad2 Signaling Pathway*

**Figure 4.**
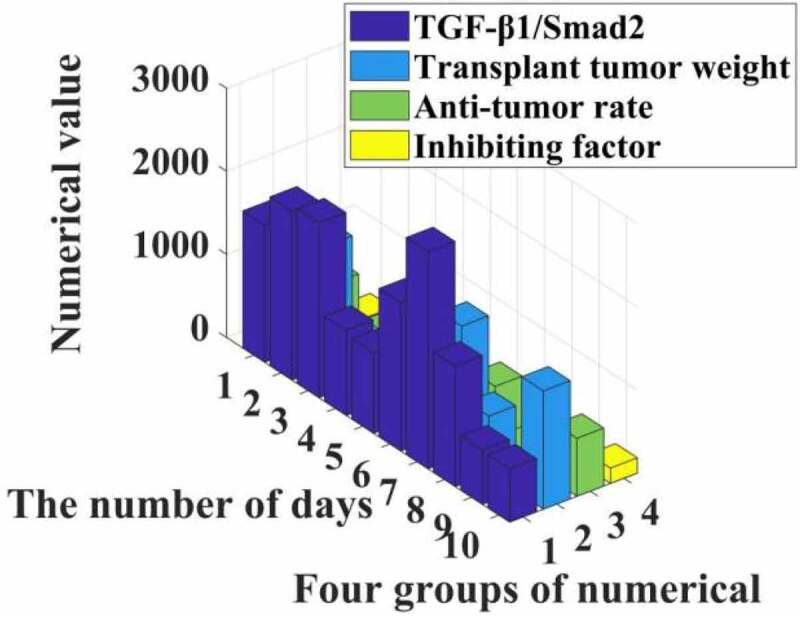
Propofol promoting the activity of TGF- 1/Smad2 signaling pathway

The experimental results show that the inhibition rate of each transplanted tumor is different (I = 446,972.709, p = 0.000). Compared with group C, the tumor inhibition rate of IGF group was negative, and the difference was statistically significant (P = 0.000). The tumor inhibition rates of group P, LY and P + LY were all increased (F < 0.05). Compared with the IGF group, the tumor inhibition rate of the P + IGF group increased, and the difference was statistically significant (/M).000); compared with the LY group, the tumor inhibition rate of the P + LY group increased, and the difference was statistically significant (P = 0.000). Among the six groups, the P + LY group had the highest tumor inhibition rate. The specific data are shown in Figure 4.

It can be seen from the data in Figure 4 that propofol can effectively increase the activity of the TGF-β1/Smad2 signaling pathway, resulting in a significant reduction in the proliferation of liver cancer cells, and the tumor inhibition rate can reach 35%. The experimental group shows that in rats the weight of the transplanted tumor is significantly reduced, and it can be seen that the Smad2 signaling pathway can indeed release inhibitors to reduce the proliferation of liver cancer cells.

**Figure 5.**
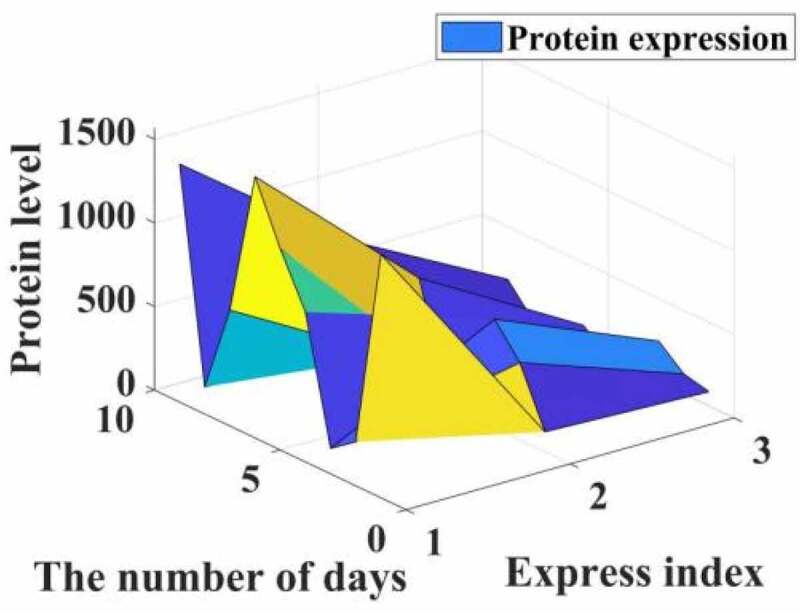
Effect of propofol on the expression of PCNA, CD34 and PATK in liver cancer patients

In addition, the surface comparison of PCNA, CD34 and PATK in the transplanted scar tissue in each group was statistically significant (I = 6535.517, f = 0.000, respectively). P = 3214.185). Compared with the control group, the expression of PCNA, CD34 and PATK protein in the transplanted tumor tissue of the IGF group was up-regulated (P < 0.05), while the expression of PCNA, CD34 and PATK protein in the transplanted tumor tissue of the P group, the LY group and the P + LY group Decrease (P < 0.05). Compared with the IGF group, the expressions of PCNA, CD34 and PATK in transplanted tumor tissues in the P + IGF group were down-regulated, and the difference was statistically significant (P = 0.000). Compared with the LY group, the expression of PCNA, CD34 and PATK in the transplanted tumor tissues of the P + LY group was down-regulated, and the difference was statistically significant (F = 0.000). In the experimental group, the expression of PCNA. CD34 and PATK in the transplanted tumor tissue of the P + LY group was the least, and the relevant data are shown in Figure 5.

It can be seen from Figure 5 that propofol can reduce the expression of PCNA, CD34, and PATK protein in patients, thereby increasing the activity and content of transforming growth factor TGF-β1 by 12% and 20%, thereby inhibiting the proliferation of liver cancer cells through the Smad2 signaling pathway. The rate reaches 10%, making the number of liver cancer cells apoptotic to increase exponentially.

Propofol has a significant inhibitory effect on the cycle of liver cancer cells. Compared with the normal cancer cell cycle, the life cycle of rat cancer cells treated with propofol drug intervention was shortened by 85.73%. In addition, propofol can greatly reduce the proliferation rate of liver cancer cells by regulating the mRNA division speed of liver cancer cells in patients with liver cancer, inhibiting and slowing down the expression intensity of mRNA Fas, Bcl-2, and Bax. Compared with the normal group, propofol can reduce its proliferation rate by 74%, and the number of liver cancer cells can be reduced by 35 percentage points compared with before treatment. Discussed and verified the effect of isopropanol on the growth of liver cancer cells and the role of TGF-β1/Smad2 signaling pathway. Experiments have shown that this program can effectively increase the activity of the TGF-β1/Smad2 signaling pathway, significantly reduce the proliferation of liver cancer cells, and the tumor inhibition rate can reach 35%. The experimental group showed that the weight of transplanted tumors in rats was significantly reduced. It can be seen that the Smad2 signaling pathway can indeed release inhibitors and reduce the proliferation of liver cancer cells.

## 5. Conclusions

This article introduces the mechanism and mechanism of propofol affecting the proliferation and apoptosis of hepatocellular carcinoma cells, studies and analyzes the mechanism of TGF-β1/Smad2 signaling pathway inhibiting the proliferation of hepatocellular carcinoma cells and its effect on hepatocellular carcinoma cells, and studies the effect of propofol in the treatment of hepatocellular carcinoma Feasibility and effectiveness. Under the action of propofol, the life cycle of liver cancer cells is shortened. Propofol can reduce the expression of PCNA, CD34, and PATK proteins in patients, thereby increasing the activity and content of transforming growth factor TGF-β1 by 12% and 20%, respectively, thereby inhibiting the proliferation rate of liver cancer cells by 10% through the Smad2 signaling pathway. The number of apoptosis of liver cancer cells increased exponentially.
